# Artificial Intelligence in Thermal Ablation: Current Applications and Future Directions in Microwave Technologies

**DOI:** 10.3390/biomimetics10120818

**Published:** 2025-12-05

**Authors:** Kealan Westby, Daniel Westby, Kevin McKevitt, Brian M. Moloney

**Affiliations:** 1 Blackrock Health Galway Clinic, H91 HHT0 Galway, Ireland; 2 Department of Radiology, University Hospital Limerick, St Nessans Rd, Dooradoyle, V94 F858 Limerick, Ireland; 3 Graduate Entry Medical School, Faculty of Education and Health Sciences, University of Limerick, V94 T9PX Limerick, Ireland

**Keywords:** artificial intelligence, thermal ablation, microwave ablation, interventional oncology, machine learning, radiomics, treatment planning, segmentation, image-guided therapy, clinical translation

## Abstract

Artificial intelligence (AI) is increasingly shaping interventional oncology, with growing interest in its application across thermal ablation modalities such as radiofrequency ablation (RFA), cryoablation, high-intensity focused ultrasound (HIFU), and microwave ablation (MWA). This review characterises the current landscape of AI-enhanced thermal ablation, with particular emphasis on emerging opportunities within MWA technologies. We examine how AI-driven methods—convolutional neural networks, radiomics, and reinforcement learning—are being applied to optimise patient selection, automate image segmentation, predict treatment response, and support real-time procedural guidance. Comparative insights are provided across ablation modalities to contextualise the unique challenges and opportunities presented by microwave systems. Emphasis is placed on integrating AI into clinical workflows, ensuring safety, improving consistency, and advancing personalised therapy. Tables summarising AI methods and applications, a conceptual workflow figure, and a research gap analysis for MWA are included to guide future work. While existing applications remain largely investigational, the convergence of AI with advanced imaging and energy delivery holds significant promise for precision oncology. We conclude with a roadmap for research and clinical translation, highlighting the need for prospective validation, regulatory clarity, and interdisciplinary collaboration to support the adoption of AI-enabled thermal ablation into routine practice.

## 1. Introduction

The integration of artificial intelligence (AI) into interventional oncology represents a critical frontier in the advancement of personalised, image-guided therapies. Thermal ablation techniques—including radiofrequency ablation (RFA), cryoablation, high-intensity focused ultrasound (HIFU), and microwave ablation (MWA)—are increasingly used to treat a variety of malignant and benign conditions, particularly in hepatic, renal, and pulmonary tumours [1,2,3]. These minimally invasive therapies rely on accurate imaging, anatomical interpretation, and intra-procedural decision-making, making them natural candidates for AI enhancement [4].

Thermal ablation is an example of biomimetic principles applied in the clinical setting through the use of controlled thermal energy for accurate tumour cell death and subsequent cell repair. The assimilation of AI into oncological thermal ablation demonstrates the progressive evolution of biomimetics in modern times, especially through the integration of artificial neural networks (ANN). Combining ANN with ablative therapies facilitates the delivery of more personalised patient care [5]. In this context, AI systems emulate biological learning processes by recognising patterns in imaging and procedural data, adapting predictions to complex anatomical environments in a manner analogous to biological optimisation. This convergence of thermal biophysics and adaptive computational learning represents a modern extension of biomimetic design within image-guided intervention.

AI, broadly defined as the simulation of human intelligence by computational systems, has demonstrated considerable promise in radiology and image-guided intervention. It encompasses diverse methods including machine learning (ML), deep learning (DL), reinforcement learning (RL), and radiomics—all of which have been explored to improve diagnosis, segmentation, treatment planning, and outcome prediction [6,7,8].

In thermal ablation, AI offers the potential to overcome longstanding challenges: from predicting ablation volume and recurrence risk to improving real-time decision-making and post-procedural assessment [9]. Among thermal techniques, MWA is uniquely positioned to benefit from AI, due to its controllable energy delivery, predictable biophysics, and increasing use in complex anatomies [10,11].

This review provides a comprehensive overview of current and emerging AI applications across thermal ablation modalities, with a focused analysis of MWA. Moving forward, the integration of AI with ablative therapies has the potential to evolve into a standard-of-care adjunct for a cohort of oncological patients. Advancing ANN creates a platform to deliver real-time data to achieve the best outcomes. We highlight key opportunities, describe evolving clinical use cases, and propose a translational roadmap to bring AI-enabled ablation into routine interventional radiology practice with particular relevance to the field of biomimetics.

### Search Strategy and Study Selection

This narrative review draws on a structured yet flexible search of the PubMed, Scopus, and Google Scholar databases, using combinations of keywords related to “microwave ablation,” “thermal ablation,” and “artificial intelligence,” including specific terms such as “machine learning,” “deep learning,” “radiomics,” and “image-guided intervention.” Additional studies were identified by screening reference lists and through citation tracking. Peer-reviewed articles focusing on AI applications in ablation planning, image analysis, procedural guidance, or outcome prediction were prioritised. Conference abstracts without full texts, non-English publications, and studies unrelated to image-guided therapy were excluded. As this work is a narrative review, formal PRISMA methodology was not applied.

## 2. Background—Thermal Ablation and Microwave Technologies

Thermal ablation is an established component of modern interventional oncology, offering minimally invasive, organ-sparing treatment for primary and metastatic tumours. Ablative therapies induce irreversible cell death by applying focused thermal energy—either heating or freezing—under imaging guidance. This controlled application of heat or cold reflects core biomimetic principles by replicating predictable patterns of thermal injury and repair observed in biological systems. The most widely employed thermal modalities include:

1. Radiofrequency ablation (RFA), which generates resistive heating via alternating current applied through electrode probes [12]; however, its performance is limited by impedance-related heating variability, reduced efficacy near large vessels, and smaller achievable ablation zones.
2. Cryoablation, which causes tissue necrosis through intracellular ice formation and vascular injury induced by freeze–thaw cycles [13]; but it requires real-time monitoring of the ice ball, carries a risk of cryoshock, and may have difficulty achieving complete margins in highly vascular organs.
3. High-intensity focused ultrasound (HIFU), a non-invasive modality that delivers focused acoustic energy to generate localised coagulative necrosis [14]; although its efficacy is constrained by long treatment times, sensitivity to patient motion, and acoustic shadowing from bone or gas.
4. Microwave ablation (MWA), which uses electromagnetic radiation—typically at 915 MHz or 2.45 GHz—to excite water molecules and produce rapid, homogeneous heating [10,11]. Its limitations include potential difficulty predicting ablation shape in heterogeneous tissues and the need for precise antenna positioning to avoid collateral heating.

Among these, MWA has gained increasing popularity due to its technical advantages: faster heating times, larger and more spherical ablation zones, and reduced susceptibility to tissue desiccation or the “heat-sink effect” caused by adjacent vasculature [15,16]. These benefits stem from its mechanism of electromagnetic heating, which—unlike the conductivity-dependent energy transfer in RFA—provides more predictable performance across varying tissue types. These characteristics are especially advantageous in the treatment of hepatocellular carcinoma (HCC), pulmonary nodules, and renal masses [17].

Technical advances in antenna design, cooling systems, and power modulation have further optimised energy delivery, procedural consistency, and safety [10]. However, despite these advancements, MWA outcomes may still vary due to local perfusion differences, tissue composition, and antenna orientation. However, challenges remain in pre-procedural planning, accurate prediction of ablation margins, and intra-procedural visualisation of the ablation zone—particularly in deep or poorly perfused lesions [2,18]. Conventional planning tools, such as manufacturer ablation charts or operator estimation, often fail to account for these patient-specific anatomical and biophysical variables. Furthermore, CT and ultrasound may inadequately depict the evolving ablation zone early during treatment, contributing to uncertainty in achieving complete margins.

These are the precise areas where artificial intelligence offers unique value. By learning from large imaging and procedural datasets, AI systems can augment operator expertise, reduce variability, and tailor therapy to patient-specific anatomy and pathology. The remainder of this review explores how these capabilities are being integrated into current thermal ablation workflows, with a particular focus on MWA as a case study in clinical translation.

## 3. Overview of Artificial Intelligence in Image-Guided Therapy

Artificial intelligence (AI) refers to a suite of computational technologies that enable machines to replicate aspects of human cognition, including learning, reasoning, and pattern recognition. In medical imaging and image-guided therapy, AI has rapidly evolved from experimental prototypes to clinically impactful tools [6,19,20].

Its adoption has been accelerated by increasing computational power, large annotated imaging datasets, and improvements in algorithmic efficiency, enabling real-time or near–real-time applications in procedural settings.

The foundation of AI in healthcare lies in Machine Learning (ML)—algorithms that learn patterns from labelled data to make predictions or classifications. Shallow ML methods (e.g., random forests, support-vector machines, logistic regression) are often advantageous when datasets are small or features are hand-crafted, offering greater interpretability and requiring fewer computational resources. A subset of ML, Deep Learning (DL), uses multi-layered neural networks to extract hierarchical features, with convolutional neural networks (CNNs) proving especially powerful for image analysis tasks such as segmentation, classification, and object detection [21,22,23]. DL models typically achieve higher accuracy than shallow ML—such as Dice similarity coefficients > 0.90 for tumour segmentation or AUROC values > 0.85 for outcome prediction—but require larger datasets and remain less interpretable. In image-guided therapy, DL is particularly valuable due to its ability to handle complex anatomical variability and high-dimensional imaging data. Other techniques increasingly used in procedural environments include:

Radiomics: The extraction of high-dimensional quantitative features from imaging data to uncover patterns predictive of pathology, prognosis, or response [8,24]. Radiomics is especially relevant to ablation therapy, where tumour heterogeneity and peritumoral characteristics influence treatment response.

Reinforcement learning (RL): Algorithms that learn optimal decision policies through interaction with an environment, with promising applications in robotic guidance and ablation optimisation [20,25]. RL is uniquely suited to procedural tasks that require sequential decision-making, such as trajectory planning or adaptive energy modulation.

Natural language processing (NLP): Applied to radiology reports and operative notes to extract structured data from free text [26]. NLP increasingly supports data curation for AI model training and facilitates automated auditing of ablation outcomes in clinical practice.

AI applications in image-guided therapy are being explored across a range of tasks:

1. Patient selection and outcome prediction: AI models have been developed to predict suitability for ablation, recurrence risk, and survival, often outperforming conventional scoring systems [27,28,29]. These models integrate imaging, clinical variables, and laboratory data, supporting personalised treatment planning.
2. Image segmentation and registration: DL enables rapid and accurate segmentation of tumours, vessels, and organs-at-risk from CT, MRI, and ultrasound [21,30]. This is foundational for pre-treatment planning and intraoperative navigation. Accurate segmentation also supports simulation-based decision tools and automated margin assessment.
3. Ablation planning and simulation: AI-driven models simulate thermal spread, predict ablation zone morphology, and optimise energy delivery based on patient-specific anatomy [31,32]. These tools address limitations of traditional reference charts by accounting for perfusion, tissue composition, and probe configuration.
4. Intra-procedural monitoring: CNNs and real-time image fusion algorithms are being trained to monitor ablation progression and detect complications as procedures unfold [33,34]. By providing dynamic feedback, these systems may help operators achieve complete margins while minimising collateral injury.
5. Post-procedural assessment: Radiomics and DL models can evaluate completeness of ablation, predict residual tumour, and support longitudinal surveillance [35,36,37]. Such tools may reduce reliance on invasive biopsy and enable earlier identification of treatment failure.

These capabilities, while at varying levels of clinical maturity, lay the groundwork for significant advances in precision interventional oncology. In the next section, we examine how these AI tools are currently being applied across different thermal ablation modalities.

## 4. Current AI Applications in Thermal Ablation

AI technologies are increasingly being integrated into various stages of the thermal ablation workflow, from patient selection to post-treatment evaluation. Building on the general AI methods outlined in Section 3, this section focuses specifically on their deployment within the thermal ablation workflow. While many tools remain investigational, the growing body of literature suggests significant potential to enhance clinical precision, consistency, and outcomes. These developments reflect a broader trend toward data-driven decision support in interventional oncology, where procedural variability and patient-specific anatomical differences present persistent challenges.

### 4.1. Patient Selection and Outcome Prediction

ML models have been trained to identify patients most likely to benefit from ablation based on imaging, clinical variables, and laboratory data [38,39]. In hepatocellular carcinoma (HCC), radiomics-based nomograms have predicted recurrence and survival following RFA or MWA with higher accuracy than traditional staging systems [40,41]. These tools are particularly valuable in heterogeneous diseases such as HCC, where conventional models may not fully capture tumour biology or microenvironmental features.

Integrating AI into multidisciplinary decision-making frameworks may help refine treatment allocation between surgical, ablative, or systemic options—especially in borderline or high-risk cases [42]. Such integration supports personalised therapy selection and may improve consistency across institutions and operators.

### 4.2. Automated Image Segmentation

Segmentation of tumours and organs-at-risk is foundational for planning and assessing thermal ablation. DL, particularly CNNs, has enabled automated segmentation in CT and MRI with Dice similarity coefficients often exceeding 0.9 in liver, kidney, and lung datasets [38,39]. Accurate segmentation reduces operator workload and supports downstream tasks such as ablation simulation and longitudinal treatment assessment.

In real-time workflows, such as intra-procedural ultrasound or CT, segmentation models are being adapted for rapid inference speeds and interactive use [43]. Some studies report successful use of AI to segment the ablated zone itself during or immediately after MWA [44]. Real-time ablation zone segmentation may help detect undertreated regions early, thereby reducing the need for repeat interventions.

### 4.3. Ablation Planning and Simulation

AI is being applied to simulate the expected ablation volume based on probe position, power settings, and tissue properties. These tools are especially relevant for MWA, where thermal spread depends on a combination of electromagnetic field distribution, tissue conductivity, and perfusion [45]. This modelling addresses a major limitation of conventional manufacturer-generated charts, which often fail to account for patient-specific anatomical variability.

Several models integrate finite element analysis with AI to provide fast and patient-specific predictions of ablation zones, enabling procedural optimisation before treatment [32,46]. Early efforts have demonstrated improved correlation between simulated and actual outcomes when AI-enhanced planning is used. As these systems mature, they may help standardise ablation strategies across operators with differing levels of experience.

### 4.4. Intra-Procedural Guidance and Real-Time Feedback

Reinforcement learning and CNNs have been explored for needle trajectory planning, offering intelligent pathfinding in anatomically complex or high-risk areas [47]. Real-time feedback loops, trained on procedural imaging, are also being developed to track thermal lesion expansion, helping operators terminate ablation when sufficient margins are achieved [48].

AI-enhanced image fusion—especially combining CT/MRI with real-time ultrasound—has improved target localization and antenna placement, particularly in mobile or obscured lesions [49]. These technologies are especially useful in lesions poorly visualised on ultrasound alone or subject to respiratory motion.

### 4.5. Post-Procedural Assessment

After ablation, it is often challenging to distinguish residual tumour from benign post-treatment changes on imaging. AI tools have shown promise in detecting incomplete ablation or early recurrence on follow-up imaging [50]; classifying radiological changes in the ablation zone using radiomics or supervised learning [51]; and predicting long-term outcomes based on post-ablation imaging and early laboratory trends [52]. These applications may reduce the need for invasive biopsy or unnecessary repeat ablations and improve surveillance protocols. Furthermore, automated post-treatment assessment has the potential to standardise reporting and reduce interobserver variability, supporting more consistent patient follow-up.

## 5. Comparative Overview—Applications Across Thermal Modalities

Although AI tools are being developed across all thermal ablation modalities, the degree of integration, maturity, and validation varies. Where Section 4 examined AI applications along the procedural workflow, this section provides a modality-specific comparison to highlight how these technologies differ across RFA, cryoablation, HIFU, and MWA. Differences in energy delivery, imaging compatibility, and procedural control influence how AI is applied and what challenges it aims to solve in each modality. Recognising these distinctions helps identify where AI can provide the most meaningful clinical benefit. A summary of current AI applications across RFA, cryoablation, HIFU, and MWA is provided in Table 1.

### 5.1. Radiofrequency Ablation (RFA)

RFA has the longest clinical history among thermal techniques, and therefore the most available data for model training. AI applications focus on outcome prediction in HCC and metastatic liver disease, often via radiomics-based models [33,53]; CT/MRI-based tumour segmentation to support treatment planning and evaluation [52]. Several studies have also explored AI models for predicting incomplete ablation near vessels, where the heat-sink effect is most pronounced, and shallow machine-learning classifiers have been used to stratify recurrence risk based on tumour morphology, perfusion, and patient-level clinical factors [54]. CNN-based segmentation methods have demonstrated Dice coefficients > 0.85 for liver tumours in RFA datasets, supporting semi-automated planning and post-ablation zone assessment [44]. However, the dependence of RFA on tissue conductivity introduces variability that may limit the precision of AI-driven predictive modelling.

### 5.2. Cryoablation

Cryoablation poses distinct challenges due to the need for real-time ice ball monitoring, often with MRI or ultrasound. AI contributions include enhanced visualisation and segmentation of the ice ball on ultrasound and MR thermometry and risk prediction of incomplete ablation based on anatomical and procedural parameters [55]. Recent work has assessed AI-assisted ice-ball boundary tracking using CNNs trained on multimodal datasets, improving reproducibility in regions where conventional ultrasound suffers from acoustic shadowing [56]. In prostate cryoablation, AI-driven registration between planning and intra-procedural imaging has improved needle-guidance accuracy and ice-ball coverage prediction [56]. Improved delineation of the ice margin is particularly valuable given the limitations of conventional ultrasound at low-contrast interfaces.

### 5.3. High-Intensity Focused Ultrasound (HIFU)

HIFU is particularly amenable to AI due to its non-invasive nature and compatibility with advanced image feedback. CNNs are used to predict focal heating zones and optimise treatment paths [57]; AI-driven control systems modulate energy delivery imaging [58]. ML models have also been used to forecast tissue displacement and acoustic propagation, supporting dynamic compensation for respiratory motion and heterogeneous tissue compositions [59]. In uterine fibroid and bone metastasis treatments, hybrid radiomics–DL frameworks have been developed to predict treatment response and streamline patient selection for HIFU therapy [60]. Real-time AI-based thermometry and adaptive beam steering may further enhance precision in this highly image-dependent modality.

### 5.4. Microwave Ablation (MWA)

MWA benefits from its predictability and energy control. AI-enhanced strategies include DL models that simulate ablation zones based on antenna type and tissue conductivity; Tumour and margin segmentation in liver, kidney, and lung applications; Use of reinforcement learning to plan antenna trajectories in high-risk locations [1,61]. Recent MWA-focused studies have evaluated DL-based thermal field prediction using ultrasound texture and features, achieving improved agreement between predicted and observed ablation geometries [62]. Software-based margin-assessment tools have also successfully shown that with 3D imaging processing, margins can be significantly reduced whilst maintaining clear margins [63]. The reproducible biophysics of MWA make it particularly suited to AI-guided planning and optimisation.

**Table 1:** AI Applications Across Thermal Ablation Modalities.

Modality	Planning	Segmentation	Real-Time Guidance	Post Procedural Assessment	Techniques
RFA	✓✓	✓✓✓	✓	✓✓✓	Radiomics, CNNs, ML
Cryoablation	✓	✓✓	✓✓✓	✓✓	CNNs, Decision Trees
HIFU	✓✓	✓	✓✓✓	✓✓	CNNs, Reinforcement Learning, DL
MWA	✓✓✓	✓✓✓	✓✓	✓✓✓	CNNs, Radiomics, Biothermal AI

✓ = early development; ✓✓ = emerging application; ✓✓✓ = active or validated use.

## 6. Focus on Microwave Ablation—Integration and AI Opportunities

**Figure 1:**
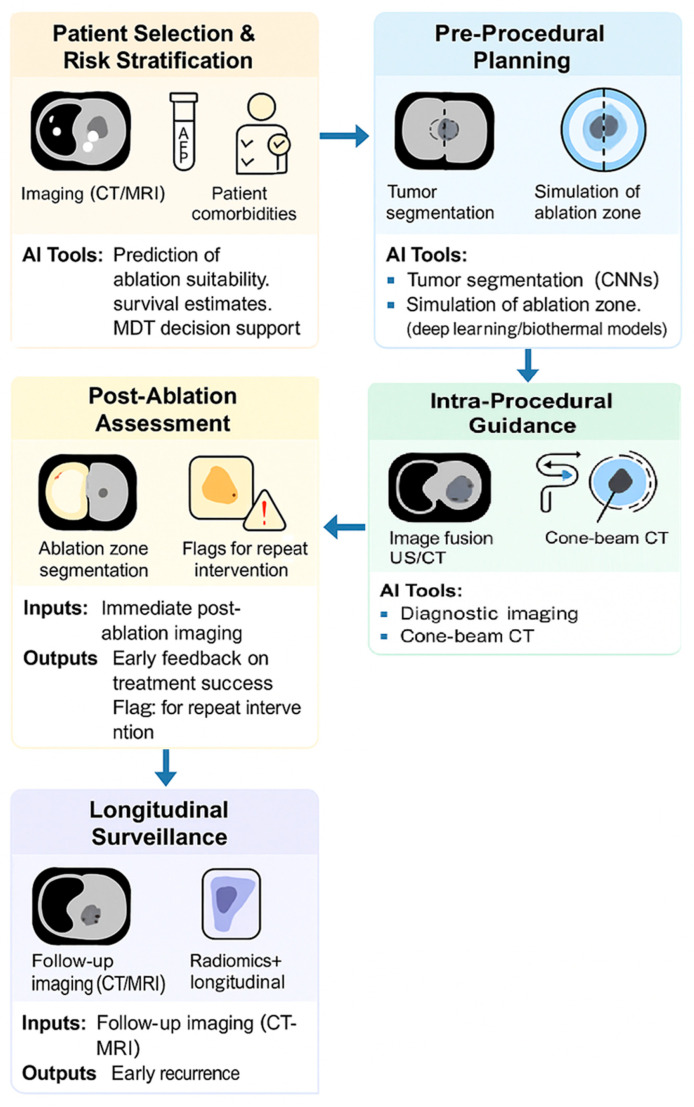
This AI-enhanced workflow mirrors existing clinical pathways but introduces data-driven intelligence at each decision point. It enables adaptive therapy, precision margin control, and longitudinal risk prediction, all aligned with current interventional radiology practice.

MWA has emerged as a preferred modality in many clinical settings due to its rapid, uniform heating and reduced susceptibility to perfusion-mediated heat loss. It is increasingly used in hepatic, renal, and pulmonary interventions, especially in patients who are not surgical candidates or in tumours near large vessels [64,65]. With increasing procedural volume and the availability of large imaging datasets, MWA is well-positioned for AI integration. An overview of a potential AI-enhanced workflow for MWA is illustrated in Figure 1.

### 6.1. Unique Technical Features Supporting AI

MWA offers several biophysical advantages that synergize with AI methods; predictable energy deposition allows thermal simulations to be trained on consistent outcomes [66]; Shorter ablation times provide clearer cause–effect relationships for training ML models [67]; Electromagnetic energy propagation can be mathematically modelled and integrated into AI systems for planning and intra-procedural control [45]. Additionally, its relative insensitivity to tissue impedance reduces interpatient variability, strengthening the reliability of AI-based prediction. These factors make MWA a strong candidate for AI-driven optimisation, particularly in simulation, margin estimation, and procedural guidance. These technical characteristics also align with biomimetic principles, as AI models learn from repeated exposure to similar biophysical patterns in a manner analogous to biological systems adapting through experience. By capturing the relationship between energy deposition, tissue response, and spatial constraints, AI-enabled MWA systems effectively replicate the adaptive optimisation strategies observed in natural processes.

### 6.2. AI-Enabled Simulation of Ablation Zones

Several studies have developed DL models that simulate the size and shape of ablation zones based on input parameters such as antenna type, power level, tissue perfusion, and anatomical context [67,68]. These models can help physicians predict the extent of necrosis before the procedure; adjust ablation parameters to improve margin coverage; and reduce the need for multiple overlapping ablations [61]. Recent MWA-specific DL models have demonstrated promising results: for instance, a U-Net trained on pre-procedure CT, applicator parameters and position accurately predicted post-treatment ablation zones in lung MWA, showing improved Dice scores and target registration error ≤ 2 mm in a substantial proportion of cases, outperforming vendor estimates [67]. These results highlight the feasibility of patient-specific ablation forecasting with clinically meaningful precision. As simulation tools mature, they may support more consistent planning across operators and tumour types.

### 6.3. Integration with Pre- and Intra-Procedure Imaging

CNN-based segmentation of MWA targets and ablation zones from CT and MRI has demonstrated high accuracy and efficiency [61]. Algorithms can automate tumour contouring for planning, estimate optimal antenna insertion paths, and identify zones of potential under-treatment in real-time [43,69]. Emerging CBCT-integrated MWA protocols, combining fusion imaging and ablation-volume prediction, have demonstrated improved intra-procedural visualisation and appear to support margin assessment in clinical practice [70]. To date, however, no published study has reported fully automated CBCT-based ablation-zone segmentation with validated performance metrics. Intra-procedural use of AI—especially on cone-beam CT or fusion ultrasound—remains under development but is highly promising for dynamic treatment adaptation. AI-enabled intra-procedural margin assessment may help address the longstanding challenge of visualising evolving ablation zones during treatment.

### 6.4. Clinical Prediction Models for MWA Outcomes

Several studies have employed radiomics and ML models to predict local recurrence, overall survival, or complication risk following MWA, particularly in HCC and colorectal liver metastases [71]. In recent radiomics-based analyses, predictive models for early recurrence after MWA have reported AUC values between 0.80 and 0.88, outperforming conventional scoring systems and enhancing MDT risk stratification [72]. These tools are increasingly being integrated into treatment decision platforms, enabling personalised risk stratification and supporting MDT discussions. Their ability to combine imaging, procedural, and clinical data positions them well for use in longitudinal care pathways.

## 7. Research Gap Analysis—AI in Microwave Ablation

Despite promising early results, AI integration in MWA remains largely preclinical or confined to retrospective datasets. Several key limitations and unmet needs must be addressed to transition AI tools from research to routine clinical practice. These gaps span technical, operational, and regulatory domains, each representing a critical barrier to reliable deployment. A summary of these key research gaps and corresponding proposed actions is presented in Table 2.

### 7.1. Technical Gaps

Limited prospective validation: Most AI studies in MWA are retrospective, with small sample sizes and heterogeneous imaging protocols [73]. This limits generalisability and reduces confidence in model performance, particularly for ablation-zone prediction and early recurrence detection. Prospective multicentre datasets with standardised acquisition parameters are needed to generate reproducible evidence.

Lack of standardised datasets: No large-scale, annotated, open-access datasets exist specifically for MWA imaging or procedural metadata [74]. This absence hampers algorithm development by restricting training volumes, preventing external validation, and contributing to overfitting—especially for DL models requiring thousands of cases to achieve stable performance.

Poor interpretability: DL models—particularly CNNs—are often “black boxes”, which may hinder clinical trust and regulatory acceptance [75]. Explainable AI (XAI) methods remain underdeveloped in the MWA domain, and current studies rarely evaluate whether model decisions align with known thermal biophysics or accepted procedural heuristics.

### 7.2. Clinical and Operational Gaps

Insufficient integration with ablation consoles: Current AI tools function in research silos and are rarely embedded in manufacturer software or navigation platforms [76]. Without direct integration into ablation workstations, real-time decision support (e.g., margin estimation, antenna adjustment suggestions) cannot meaningfully influence clinical care.

Workflow disruption risk: In busy interventional suites, AI systems must offer real-time performance and seamless integration without delaying procedures [77]. Most existing algorithms require offline processing, cloud computation, or manual preprocessing steps, making them impractical for intra-procedural use. Edge computing and on-device inference will likely be required for safe clinical deployment.

Unclear regulatory pathway: The absence of FDA/EMA-cleared AI tools specifically for MWA highlights challenges in validation, safety assurance, and clinical utility demonstration [78]. Thermal ablation presents unique regulatory complexities because models may influence procedural decisions, such as ablation duration or antenna repositioning, placing them closer to “real-time decision-support” devices subject to stricter oversight.

**Table 2:** Key Research Gaps and Proposed Actions for AI Integration in Microwave Ablation.

Domain	Gap Description	Suggested Action
Data availability	Lack of large, annotated datasets for MWA segmentation, planning, and outcomes	Develop multi-centre open-source datasets
Clinical validation	Most tools validated only retrospectively	Design prospective, multi-arm clinical trials
Interpretability	“Black-box” models hinder clinician trust	Prioritise explainable AI (XAI) approaches
Real-time utility	Intra-procedural AI models are rarely fast enough	Invest in low-latency, edge-deployed AI
Regulatory integration	No microwave-specific AI models currently cleared by FDA/EMA	Partner with device manufacturers early
System integration	Standalone AI tools may not interface with ablation hardware or navigation systems	Standardise APIs for procedural interoperability

Addressing these gaps will require collaboration between radiologists, engineers, manufacturers, and regulators. A coordinated effort to expand high-quality datasets, validate models prospectively, develop interpretable algorithms, and embed AI into procedural environments is essential for safe and effective adoption. In the following section, we outline a roadmap to clinical translation, based on these insights.

## 8. Future Directions—Roadmap for Clinical Translation

To realise the full potential of artificial intelligence in MWA, a strategic, interdisciplinary roadmap is essential. Future progress must focus not only on algorithmic performance, but also on clinical validation, regulatory alignment, and seamless workflow integration. A coordinated approach involving clinicians, engineers, device manufacturers, computer scientists, regulators, and hospital IT governance teams is necessary to ensure safe and effective deployment.

### 8.1. Prospective Multicenter Validation

Most current AI studies in thermal ablation are retrospective and single centre, limiting generalizability. Large-scale, prospective validation studies—using standardised imaging protocols and outcome measures—are essential to demonstrate clinical benefit. Collaborative registries and consortia will be critical in overcoming data fragmentation and enabling model reproducibility [79]. A practical next step is the establishment of multi-centre datasets with harmonised imaging acquisition parameters, standardised annotations, and clearly defined endpoints such as residual disease, recurrence, and procedural complications. Such initiatives would enable head-to-head comparison of algorithms and permit robust external validation—key requirements for regulatory clearance.

### 8.2. Embedding AI into Ablation Ecosystems

AI tools must be embedded into navigation platforms, ablator consoles, and electronic health records (EHRs) to deliver value at the point of care. APIs and plug-ins should allow seamless use of AI without disrupting procedural flow. Manufacturers are increasingly exploring software–hardware integration, and early collaboration between developers and vendors is encouraged [80]. Implementation will require defined interface standards (e.g., DICOM, HL7/FHIR), real-time device communication protocols, and support for edge computing to ensure latency-free performance during procedures. Hospitals should prioritise compatibility testing and co-design sessions between clinical teams, device engineers, and software developers to streamline integration.

### 8.3. Emphasis on Explainability and Trust

Explainable AI (XAI) methods—such as heatmaps, feature importance visualisation, and model transparency dashboards—can enhance physician trust and support regulatory compliance [81]. Especially in high-stakes procedures like thermal ablation, clinicians must understand why an AI model made a particular suggestion. Incorporating XAI into ablation platforms will require user-facing visual tools that quickly communicate model rationale during planning and post-procedural assessment. Training curricula should include case-based examples of XAI interpretation to build confidence among interventional radiologists.

### 8.4. Regulatory Pathways and Clinical Implementation

AI-driven technologies for ablation must meet rigorous standards of safety, transparency, and utility. Regulatory bodies like the FDA and EMA have issued evolving frameworks for software as a medical device (SaMD), but thermal ablation-specific tools are still in early development [78]. Early engagement with regulators may streamline approval pathways and reduce implementation delays. Institutions planning to adopt AI for ablation should consider involvement of institutional AI governance committees, defined standards for performance auditing and have clear medico-legal boundaries for AI-assisted decisions. Key regulatory milestones include consistent external validation, cybersecurity evaluation, version-control documentation, and mechanisms for continuous learning that comply with regulatory “locked” or “adaptive” model classifications. Collaboration with manufacturers early in model development can streamline these processes.

### 8.5. Training the Interventional Radiologist of the Future

As AI tools mature, radiology education must adapt to include understanding of AI principles and limitations; Critical appraisal of model performance; and workflow integration and troubleshooting. Radiologists equipped with these skills will be better positioned to act as AI interpreters and gatekeepers, ensuring safe and appropriate application in clinical practice [73]. Training frameworks should include simulation-based modules for AI-assisted ablation planning, interpretation of real-time AI alerts, and familiarity with XAI tools. Formalised competency pathways—potentially aligned with subspecialty fellowship curricula—would ensure consistent skill acquisition across centres.

Overall, a staged roadmap involving evidence generation (Phase 1), technical integration (Phase 2), regulatory clearance (Phase 3), and widespread clinical adoption supported by education (Phase 4) provides a practical paradigm for translating AI in MWA from research into routine practice.

## 9. Conclusions

Artificial intelligence is rapidly transforming the field of interventional oncology, with thermal ablation emerging as a key application domain. Across modalities—RFA, cryoablation, high-intensity focused ultrasound, and especially MWA—AI tools are being developed to assist with patient selection, imaging interpretation, procedural planning, real-time monitoring, and post-treatment assessment.

MWA stands out due to its biophysical predictability, energy controllability, and growing clinical footprint. These characteristics make it particularly suitable for AI-enhanced approaches, including predictive thermal simulations, DL-based segmentation, and outcome modelling.

However, meaningful clinical translation remains limited by data silos, lack of prospective validation, and insufficient system integration. A collaborative roadmap involving clinicians, engineers, industry, and regulators is essential. Priorities include the development of shared datasets, explainable algorithms, embedded AI tools, and clear regulatory pathways.

Rather than replacing expertise, AI in MWA offers an opportunity to elevate it—supporting interventional radiologists in delivering more precise, personalised, and efficient care. By mirroring biological processes of adaptation, optimisation, and pattern recognition, AI-enhanced MWA exemplifies the application of biomimetic principles within modern interventional oncology. By bridging the gap between data-driven insight and procedural decision-making, AI has the potential to reshape the future landscape of image-guided oncology.

## Data Availability

Data is contained within the article.
